# Surveillance of carbapenem-resistant organisms using next-generation sequencing

**DOI:** 10.3389/fpubh.2023.1184045

**Published:** 2023-05-15

**Authors:** Katelin V. Gali, Rachael M. St. Jacques, Cheyanne I. D. Daniels, Allison O'Rourke, Lauren Turner

**Affiliations:** ^1^Division of Consolidated Laboratory Services, Department of General Services, Richmond, VA, United States; ^2^Division of Clinical Epidemiology, Office of Epidemiology, Virginia Department of Health, Richmond, VA, United States

**Keywords:** next-generation sequencing, antimicrobial resistance, outbreak, surveillance, carbapenem-resistant organisms

## Abstract

The genomic data generated from next-generation sequencing (NGS) provides nucleotide-level resolution of bacterial genomes which is critical for disease surveillance and the implementation of prevention strategies to interrupt the spread of antimicrobial resistance (AMR) bacteria. Infection with AMR bacteria, including Gram-negative Carbapenem-Resistant Organisms (CRO), may be acute and recurrent—once they have colonized a patient, they are notoriously difficult to eradicate. Through phylogenetic tools that assess the single nucleotide polymorphisms (SNPs) within a pathogen genome dataset, public health scientists can estimate the genetic identity between isolates. This information is used as an epidemiologic proxy of a putative outbreak. Pathogens with minimal to no differences in SNPs are likely to be the same strain attributable to a common source or transmission between cases. These genomic comparisons enhance public health response by prompting targeted intervention and infection control measures. This methodology overview demonstrates the utility of phenotypic and molecular assays, antimicrobial susceptibility testing (AST), NGS, publicly available genomics databases, and open-source bioinformatics pipelines for a tiered workflow to detect resistance genes and potential clusters of illness. These methods, when used in combination, facilitate a genomic surveillance workflow for detecting potential AMR bacterial outbreaks to inform epidemiologic investigations. Use of this workflow helps to target and focus epidemiologic resources to the cases with the highest likelihood of being related.

## Introduction

The United States Centers for Disease Control and Prevention (CDC) places Gram-negative carbapenem-resistant organisms (CRO) into the top five most urgent antimicrobial resistance threats in the United States (1). Carbapenem-resistant organisms of public health significance include Enterobacterales order organisms*, Pseudomonas aeruginosa*, and *Acinetobacter baumannii*. Identifying antimicrobial resistance (AMR) genes and disease clusters within the population is essential for preventing and controlling the spread of these pathogens. Next-generation sequencing (NGS) is key to identifying specific resistance genes and their spread through a population. Comparison of pathogens at the nucleotide level using NGS data allows for the determination of relatedness between bacterial isolates. Identifying clusters of closely related bacterial infections by genomic comparison enhances the public health response by enabling targeted intervention and infection control measures.

The Combating Antibiotic Resistant-Bacteria (CARB) initiative began in 2014 and continued with the US National Action Plan for Combating Antimicrobial-Resistant Bacteria, 2020–2025 (2). The initiative spurred the creation of the Antimicrobial Resistance Laboratory Network (ARLN) in 2016 (3). As a CDC ARLN site, Virginia's Division of Consolidated Laboratory Services (DCLS) began receiving CRO submissions in 2017 and implemented testing to identify carbapenemase-producing organisms, antimicrobial susceptibility testing, and PCR resistance gene identification.

In 2019, DCLS began sequencing a subset of Virginia CRO isolates. DCLS utilizes the State Public Health Bioinformatics (StaPH-B) Toolkit (4), a free and open-source python wrapper for various bioinformatics tools and Nextflow-based workflows, to analyze pre-defined AMR datasets. While the workflows are written in the workflow manager language Nextflow, other languages (such as Python, BASH, and JavaScript) and tools are used as well. Each workflow utilizes Docker containers, or compartmentalized tools and their associated dependencies, to produce actionable public health data. Workflows and tools that are hosted in the StaPH-B Toolkit are developed by a U.S. public health laboratory consortium (5) and are subjected to rigorous validation and verification processes. Each of the discussed bioinformatics tools included herein (except for National Center for Biotechnology Information (NCBI) Pathogen Detection) are included in the Toolkit.

Public health laboratories receiving CDC funding for CRO sequencing are required to submit sequences to NCBI Pathogen Detection (6, 7). One advantage of submission to Pathogen Detection is for broad swath surveillance for potential genetically related isolates among all reads submitted to NCBI under organism-specific umbrella BioProjects surveilled by Pathogen Detection. Adopting NGS methods for detection of clusters of AMR bacterial isolates, as well as identification of the underlying resistance mechanisms harbored, varies substantially between laboratories. While ongoing development within public health laboratories for more efficient and actionable utilization of NGS data continues, genomic comparison has proven useful in detecting and controlling outbreaks of AMR infections (8, 9). By harnessing the aforementioned services and bioinformatics software, a tiered workflow for surveillance of resistance genes and identification of potential disease clusters of these pathogens was piloted and is proposed for consideration by the broader public health community.

## Materials and methods

### Microbiology methods

Carbapenem resistance screening for organisms that have acquired a carbapenemase-producing gene begins by testing bacterial cultures for carbapenemase enzyme production using the modified Carbapenem Inactivation Method (mCIM) (10). All mCIM-positive isolates receive PCR testing using the Streck™ ARM-D β-lactamase PCR kit and antimicrobial susceptibility testing using the Sensititre™ Gram Negative MIC GN7F Plate (ThermoFisher Scientific, Waltham, Massachusetts). The Streck PCR assay detects the presence of the five most common carbapenemase genes (KPC, NDM, VIM, IMP, and OXA-48). Further genomic characterization using next-generation sequencing is performed on isolates meeting one of the following CDC ARLN criteria: (i) Enterobacterales PCR-positive for any carbapenemase gene other than, or in addition to KPC, due to the high prevalence of KPC-positive isolates in Virginia, (ii) *Pseudomonas aeruginosa* and *Acinetobacter baumannii* isolates that are PCR positive for any carbapenemase gene, including KPC, due to the low KPC-positivity for these organisms in Virginia, (iii) Enterobacterales*, P. aeruginosa*, or *A. baumannii* isolates with resistance or non-susceptibility to all drugs in the Sensititre panel and the submitting facility's antimicrobial susceptibility testing panel. (iv) organisms that are PCR-positive for two or more carbapenemase genes; (v) mCIM-positive and PCR-negative cultures which may harbor a novel resistance mechanism (11). Of these criteria, novel resistance is the highest priority for identification of emerging resistance factors.

### Extraction and sequencing methods

#### Manual DNA extraction

Carbapenem-resistant genomic DNA is extracted using QIAGEN QIAamp DNA Mini Kit (Qiagen, Aarhus, Denmark) from isolated bacterial colonies grown on Tryptic Soy Agar (TSA) with 5% sheep blood agar (Remel, Lenexa, Kansas) for 18–24 h at 33–37°C. The following modifications were implemented to the QIAGEN QIAamp DNA Mini Kit (12) method to obtain optimal total DNA for short-read sequencing. Cell lysis is performed in a biosafety cabinet to render the isolate no longer infectious. A loopful of isolated bacterial colonies from the TSA with 5% sheep blood agar plate is added into a labeled 1.5 mL safe-lock tube with 180 μL of ATL buffer, vortexed and pulse-centrifuged. Proteinase K (20 μL) is added to the sample and incubated at 56°C ± 1 for 1–3 h with vortexing every 20 mins. Immediately after incubation, 4 μL of RNase A (Qiagen, Aarhus, Denmark) is added to the sample, held at room temperature for 3–5 mins, followed by 200 μL of AL buffer and 200 μL of 100% ethanol (Pharmco, Brookfield, Connecticut). For quality assurance, a blank sample, or no template control (NTC) is carried throughout the extraction and sequencing procedures to assess contamination or other quality errors in testing.

Following cell lysis, samples are safely manipulated on the bench top for DNA cleanup. The entire cell lysis volume is transferred to a spin column placed in a 2 mL collection tube and centrifuged at 6,000 x g for 1 min to bind genomic DNA (gDNA) to the spin column's silica membrane. Then, following the manufacturer's protocol, the spin column is washed twice using 500 μL of AW1 and AW2 buffers at 6,000 x g for 1 min and 20,000 x g for 3 mins, respectively, and then eluted into a clean tube using 100 μL of 10 mM Tris-HCl, pH 8 (Fisher Scientific, Hampton, New Hampshire). DNA quantification post extraction is measured with the Qubit dsDNA Broad Range Assay Kit (Thermo Fisher Scientific, Waltham, Massachusetts) on a Qubit fluorometer (Thermo Fisher Scientific, Waltham, Massachusetts) to remove any samples with suboptimal concentration (≤ 5 ng/μL) from further testing.

#### Whole genome sequencing (WGS)

The number of samples per sequencing run is determined by the 500-cycle MiSeq Reagent Kit v2 (Illumina, San Diego, CA), which has a maximum output of 8.5 Gb. For optimal run quality, the total genome load for a 500-cycle cartridge is limited to 100 megabase pairs (Mbps), equivalent to up to 20 cultures with 5 Mbp genomes (13). A diverse run composition of bacterial species is selected for library preparation. However, GC-rich content organisms, such as *P. aeruginosa*, are limited to 4 to 6 samples per run to avoid bias in sample read coverage (14).

WGS of bacterial isolates includes six components: library preparation, quantification, optional fragment analysis, normalization, denaturation, and loading (15). Samples are prepared for WGS using the Illumina DNA Prep kit (Illumina, San Diego, CA) with an average of 100–500 ng of input gDNA per sample for a total volume of 30 μL. The library clean-up procedure has been modified to utilize 40. 8 μL SPB/IPB and 44.2 μl H_2_O per sample, to capture longer DNA fragments (13). Quantification and fragment analysis is recommended at the end of preparation to evaluate the quality of individual DNA and pooled libraries. The blank (NTC control) is not loaded in the final pool but is assessed for quality using the Qubit fluorometer (see below).

Individual DNA and pooled libraries are quantified using the Qubit dsDNA High Sensitivity Assay Kit (Thermo Fisher Scientific, Waltham, Massachusetts). Libraries prepared using the Illumina DNA Prep method have an average quantification value of 10 ng/μL; however, the quantification value can vary. The allowable quantification values for library blanks are ≤ 0.1 ng/μL for Qubit 2.0 and “out of range” for Qubit 3 and 4. Fragment analysis is completed using the Agilent D5000 ScreenTape kit and 4200 TapeStation System. Average fragment sizes are obtained using the region view, usually 800–1,000 bp.

Samples can be normalized individually; however, this procedure uses the pool normalization method. This method takes the pool concentration and average fragment size to calculate the molarity of DNA from the pooled libraries, molarity (nM) = [(Pool concentration ng/μL) / (660 g/mol x fragment size bp)] x 10^6^. The preferred starting library concentration for denaturation and loading is 4 nM. The formula M1V1 = M2V2 calculates the amount of pooled library required to achieve 50 μL of a 4 nM pool (200 / molarity). The volume of the pool required is then subtracted from 50 μL to determine the volume of diluent. The 4 nM pool is denatured with 0.2 N NaOH and denaturation is halted, and the 4 nM pool is further diluted using 990 μL of HTl. At this step, the denatured pool has a concentration of 20 pM and will be diluted for optimal clustering. The formula C1V1 = C2V2 is applied to calculate the amount of denatured pool required to achieve a final loading concentration of 15 pM, (20 pM) V1 = (15 pM) (1,000 mL).

DNA sequencing is performed on the Illumina MiSeq Sequencing System using the 500 cycle v2 (2 x 251) base pair sequencing chemistry. A PhiX Control v3 Library (Illumina, San Diego, CA) is helpful for troubleshooting issues with cluster density related to library preparation. The PhiX solution is denatured and diluted to match the pooled library at 15 pM and is spiked into the final pool at 1%. The denatured DNA/PhiX library pool is heated at 96°C ± 1 for 2 mins and submerged in ice for 5 mins before transferring 600 μL into the 500-cycle MiSeq cartridge.

Sample sheets are built on Local Run Manager (16). The cartridge and buffers are loaded into the instrument. MiSeq Control Software is used to start the WGS run which requires a BaseSpace account to access sequencing data for analysis. Prior to starting the run, the MiSeq will do a system check to verify the run parameters, reagent radio-frequency identification (RFID), available disk space, and internet access. Following the pre-run check, the run is started and takes ~36 h.

Post-run metrics are reviewed to assess the overall run quality. If critical run metrics pass (see Table 1), the run is accepted for initial bioinformatics analyses. Runs with quality metrics below the expected results are comprehensively reviewed for troubleshooting purposes and reloaded from library preparation. Run performance can vary depending on run composition, library preparation, and instrument errors; however, the Illumina Sequencing Analysis Viewer can be used to investigate possible solutions (17).

**Table 1:** Post run quality metrics.

**Quality metrics**	**Cluster passing filter**	**Q30 (%)**	**Q30 R1/R2 (%)**	**Cluster density (K/mm^2^)**	**Estimated yield (Gb)**	**Aligned PhiX (%)**	**PhiX error rate (%)**
Expected results	≥80	75	N/A	600–1,200	N/A	0.88–1.85	0.92–1.45

### Bioinformatics methods

#### Machine configuration

Bioinformatics analyses were performed using Amazon Web Service Elastic Cloud Computing (AWS EC2) environments with base Ubuntu 18.04 Bionic image virtual machines (VMs) with a T2.2xlarge image (8 vCPUs, 32 GB of RAM).

#### Tredegar

The DCLS-developed and validated Tredegar pipeline was used to analyze short-read Illumina data for quality and taxonomic label verification of WGS data (18). Once sequencing run data is pushed from the MiSeq instruments to BaseSpace, the data is pulled from the cloud and analyzed on the VMs for quality control.

The following command was used for each analysis:

$ staphb-wf tredegar -o<output_directory> <path/to/reads>

After the data is pulled from BaseSpace, Tredegar is utilized to calculate the average read quality for both forward and reverse reads. Minimum data acceptability criteria include (i) *fastq* Q scores ≥ 30 for both the forward and reverse reads (r1_q and r2_q, respectively), (ii) an estimated genome length (est_genome_length) within 0.5 Mbps of the expected genome size as determined on the NCBI Genome browser (19), (iii) an estimated coverage (est_cvg) ≥ 40x (the total number of bases generated for the run divided by the assembly length estimated from the *de novo* Shovill assembly (20), (iv) assembled contig (number_contigs) < 200, and (v) the species prediction (species_prediction) by MASH (21) must match the organism determined by MALDI-TOF Mass Spectrometry. Deviation from these metrics may point to contamination, sample switching, or sequencing malfunction.

Tredegar analyses are reported to the sequencing scientist via custom-designed CSV files (Table 2). Isolates with quality metrics failing to meet the above criteria are rejected and excluded from further bioinformatics analysis. Sequences meeting quality metrics are submitted to NCBI Pathogen Detection.

**Table 2:** Example of Tredegar results with passing quality metrics.

**Sample**	**rq_1**	**r2_q**	**est_genome_length**	**est_cvg**	**number_contigs**	**species_prediction**	**subspecies_prediction**
2022EP-00093	35.26	31.45	5524279	81.33	74	Klebsiella_pneumoniae	NA
2022EP-00091	37.09	34.98	3807676	85.89	51	Acinetobacter_baumannii	NA
2022EP-00092	35.04	31.55	5398118	59.17	47	Serratia_marcescens	NA
2021EP-00104	36.91	35.32	3871668	145.61	93	Acinetobacter_baumannii	NA
2021EP-00106	36.7	34.64	3937667	105.59	100	Acinetobacter_baumannii	NA
2022EP-00007	35.69	32.16	5313287	102.98	147	Escherichia_coli	O102:H6

#### NCBI pathogen detection

Illumina sequencing reads and minimum isolate metadata (excluding patient identifiable information), are submitted to the NCBI Sequence Read Archive and CDC HAI-Seq Umbrella Project, Gram Negative Bacteria BioProject PRJNA288601 with a unique sample identification number assigned by the sequencing laboratory for sample anonymity (6). Submission to an Umbrella BioProject linked to NCBI Pathogen Detection prompts the automatic analysis of reads for integration into the Pathogen Detection Project (7, 22). In Pathogen Detection, there are two different clustering pipelines in operation. For organisms which have a whole genome multiple locus sequence type (wgMLST) scheme available, a reference wgMLST scheme is used to identify the loci and alleles in each assembled genome, and then a 25-allele cut-off is applied to identify potential cluster related isolates. The second process for organisms with less than 1,000 isolates on Pathogen Detection, or for which there is not a wgMLST scheme utilizes k-mer distances to first cluster related isolates, and then a first pass SNP analysis. Clusters are created using 50-SNP single-linkage clustering. Once clusters are created by the wgMLST or K-mer process, a reference is selected within each, assemblies are aligned against the reference, SNPs are called, and phylogenetic trees inferred. The sizes of clusters may vary from two isolates to thousands, and for each organism group isolates which do not fall within the cluster detection criteria are omitted (23). The cluster analysis process automatically starts once daily for each organism, if new data are submitted.

Pathogen Detection provides AMR gene prediction for all submitted isolates in addition to SNP distances and phylogenetic trees for clustered isolates (22, 24). An email notification alert was built to alert analysts when a submitted isolate is added into a SNP cluster on NCBI. In the DCLS surveillance workflow, NCBI provides the initial phylogenetic and cluster analysis.

#### Hickory

For analysis of a pre-defined organism dataset, the DCLS-developed Hickory (25) bioinformatics pipeline was used to determine the most appropriate reference genome within the Illumina short-read dataset via MASH (21). The Hickory pipeline takes in fastq files and generates assemblies from the data. Once the fasta files have been generated, binary sketches of the fasta files are drawn within an individual directory using MASH. The fasta file sketch, or genome sketch, with the least MASH distance from the other fasta file sketches in the directory is selected as the most appropriate reference genome. The selection of a reference genome with the least distance from the dataset is important because it increases the number of nucleotide positions available for comparative genomics, and therefore, inferences made about genomic similarity or dissimilarity of a dataset. Hickory provides the reference-free FASTA assembly file of the appropriate reference genome for each dataset analyzed. This FASTA file is then used as the reference genome during Dryad analysis. Hickory ensures a closely related reference is used for comparative genomic analysis so that the maximum number of positions can be queried.

After separating the read data by species, the following command was used for each analysis:

$ staphb-wf hickory -o<output_directory> <path/to/reads>

#### Dryad

Isolates that pass Tredegar metrics are analyzed by Dryad, a bioinformatics tool developed by the Wisconsin State Public Health Labs and validated by DCLS (26). Dryad utilizes the CFSAN SNP pipeline to determine the SNP distance between closely related samples (27). Potential AMR determinants are identified via AMRFinder Plus (22, 24).

After separating the read data by species, the following command was used for each analysis:

$ staphb-wf dryad main -cg -s -r<reference.fasta> -o <output_dir> /

–report <reads>

Dryad analyses produce SNP-distance heatmaps, phylogenetics trees, and if selected during the analysis initiation, a list of AMR gene predictions. Isolates that are ≤ 11 SNPs apart are considered “putative” outbreak clusters; bioinformaticians rely on epidemiologists and their gathered evidence to determine if an isolate is truly related. Isolates that are between 12 and 15 SNPs apart can still be considered related with enough supporting epidemiological evidence. Carbapenem-resistant isolates can be considered related at a larger SNP range than other isolates; isolates that are between 12 and 30 SNPs apart may be determined to be related with epidemiological support.

**Figure 1:**
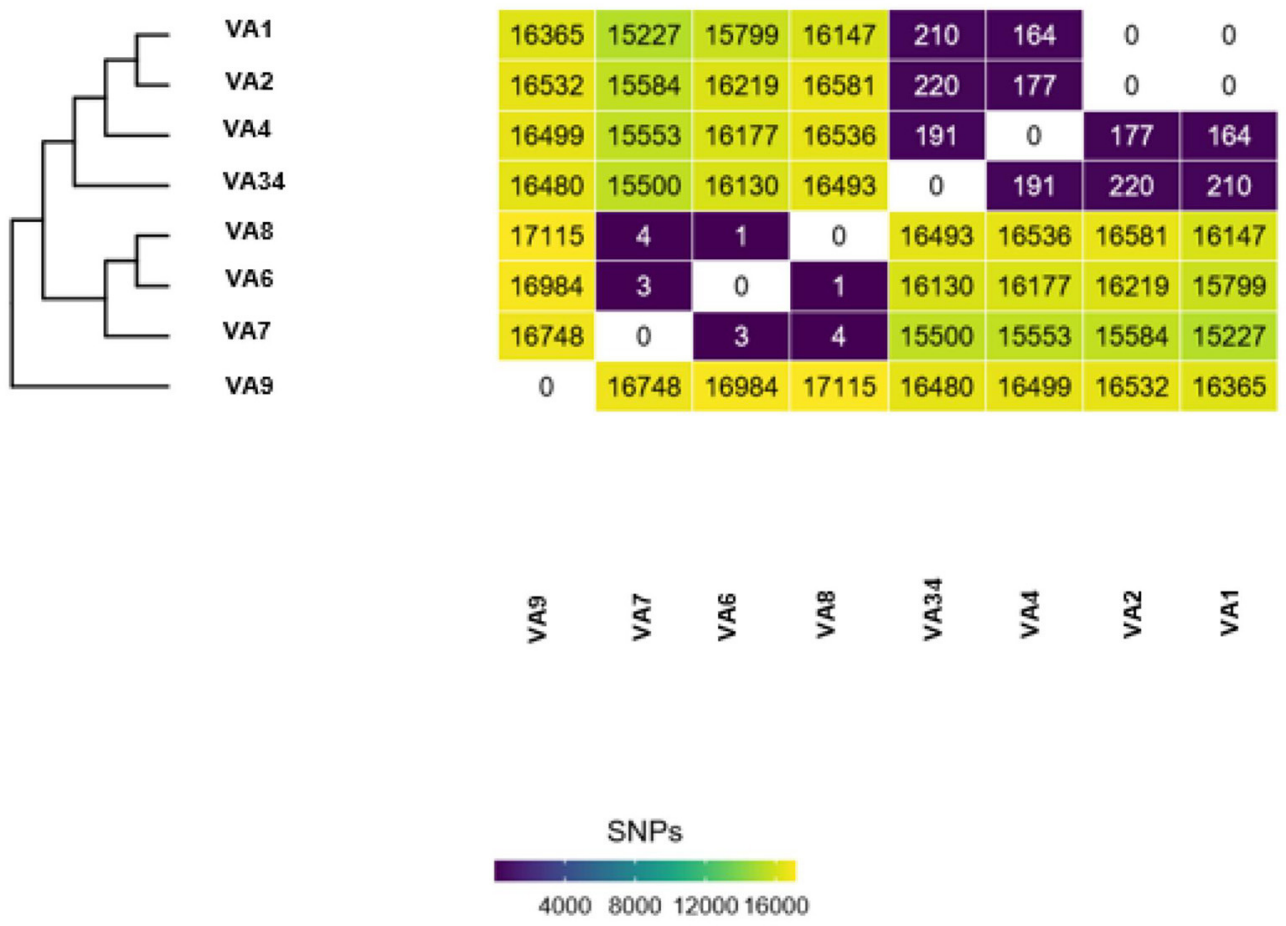
Example output of Dryad data. Each Dryad analysis produces a SNP-distance heatmap and phylogenetic tree.

Individual introduction cases are determined by the number of SNPs separating the isolates in an outbreak dataset. For example, Figure 1 shows isolates VA7, VA6, and VA8 are between 1 and 4 SNPs apart from one another. Isolates VA1 and VA2 are 0 SNPs apart from one another. This shows two putative clusters in the dataset; Group A, composed of VA7, VA6, and VA8, and Group B, composed of VA1 and VA2. These results indicate the presence of two putative outbreak clusters, or two separate introductions.

#### GAMMA

GAMMA (28), Gene Allele Mutation Microbial Assessment, is a CDC-developed bioinformatics software tool designed to analyze FASTA files to identify protein coding regions of interest. Currently, DCLS is utilizing a CDC provided custom database to elucidate hyper-virulence genes (*peg-344, iroB, iucA*, _*p*_*rmpA*, and _*p*_*rmpA2*) from sequencing assembly. GAMMA uses a Conda environment during routine analyses. GAMMA result TSV files are passed to the requesting scientists for epidemiology-report generation. Hypervirulence genes identified by GAMMA are submitted to the CDC ARLN branch.

The following command was used for each analysis:

$ GAMMA.py fasta_file custom_db.fasta output_dir

## Results

### NCBI cluster surveillance

In November 2021, DCLS began piloting a program using NCBI Pathogen Detection in a tiered surveillance method. Table 3 demonstrates the value in using NCBI Pathogen Detection as the primary step in the surveillance method. Of the 381 isolates sequenced from 2019 when CRO sequencing began until May 2022, 104 cluster notifications would have been received that include Virginia isolates. After removing clusters that only included multiple isolates from the same patient, 91 clusters would have prompted further investigation. Many of these clusters carry over from 1 year to the next due to the long-term colonization of patients and environments with resistant organisms.

**Table 3:** Isolates sequenced and NCBI clusters identified.

	**Isolates sequenced**	**NCBI clusters**	**Adjusted-removed same patient clusters**	**Adjusted-removed clusters from other years**
2019	148	39	32	32
2020	102	30	26	17
2021	119	30	28	17
2022	12	5	5	2
**Total**	**381**	**104**	**91**	**68**

Once a cluster is identified, scientists review the notification email from NCBI. The DCLS criteria for potential outbreak surveillance are more stringent (≤ 11 SNPs) compared to NCBI (≤ 50 SNPs). As mentioned previously, isolates between 12 and 30 SNPs may be included if there is epidemiologic evidence. Scientists will identify cluster isolates within 11 SNPs of each other and verify there are at least three Virginia isolates in the cluster (per epidemiologist request). Bioinformaticians use Hickory and Dryad for a more thorough investigation of the identified cluster. Dryad assesses and confirms the SNP distances between the clustered isolates using a within-dataset reference genome determined by Hickory. Both Dryad and Hickory utilize well established, open source, peer reviewed bioinformatics tools and have been validated through a rigorous state validation process. Once the Dryad pipeline confirms the SNP distance and AMR gene prediction results from NCBI Pathogen Detection, DCLS scientists build a surveillance report based on the combined wet-lab and bioinformatics results to communicate the findings to the epidemiologist.

The surveillance report includes the SNP matrix, resistance gene predictions confirmed by PCR (ex: NDM, VIM, KPC, OXA-48, or IMP), patient identification, and AST results. Since March 2022, the implementation of the NGS surveillance process has resulted in 30 communications of potential outbreak clusters. Five of these were for Enterobacterales and *Pseudomonas aeruginosa* which are provided in a surveillance report to epidemiologists at the Virginia Department of Health (VDH). The other communications were for *Acinetobacter baumannii* isolates which are of secondary priority to VDH epidemiologists and per request, cluster information is e-mailed to the epidemiologist. All NGS result reports include a disclaimer stating results are not for clinical diagnosis or patient management but are for epidemiologic purposes only. NGS results are communicated only to the health department epidemiologists.

While Dryad is a useful tool for analyzing individual outbreak datasets, the process is reliant on scientists submitting requests for known isolates. By utilizing the NCBI cluster detection pipeline, DCLS has begun to identify and analyze outbreaks both within-state and nationally. Since NCBI Pathogen Detection includes submissions from other laboratories, including other public health laboratories, cluster notifications can include DCLS isolates, and closely related isolates sequenced at other public health laboratories. Informing epidemiologists of these potential links to out-of-state isolates assists in determining possible sources of infection or enabling multi-state investigations.

**Figure 2:**
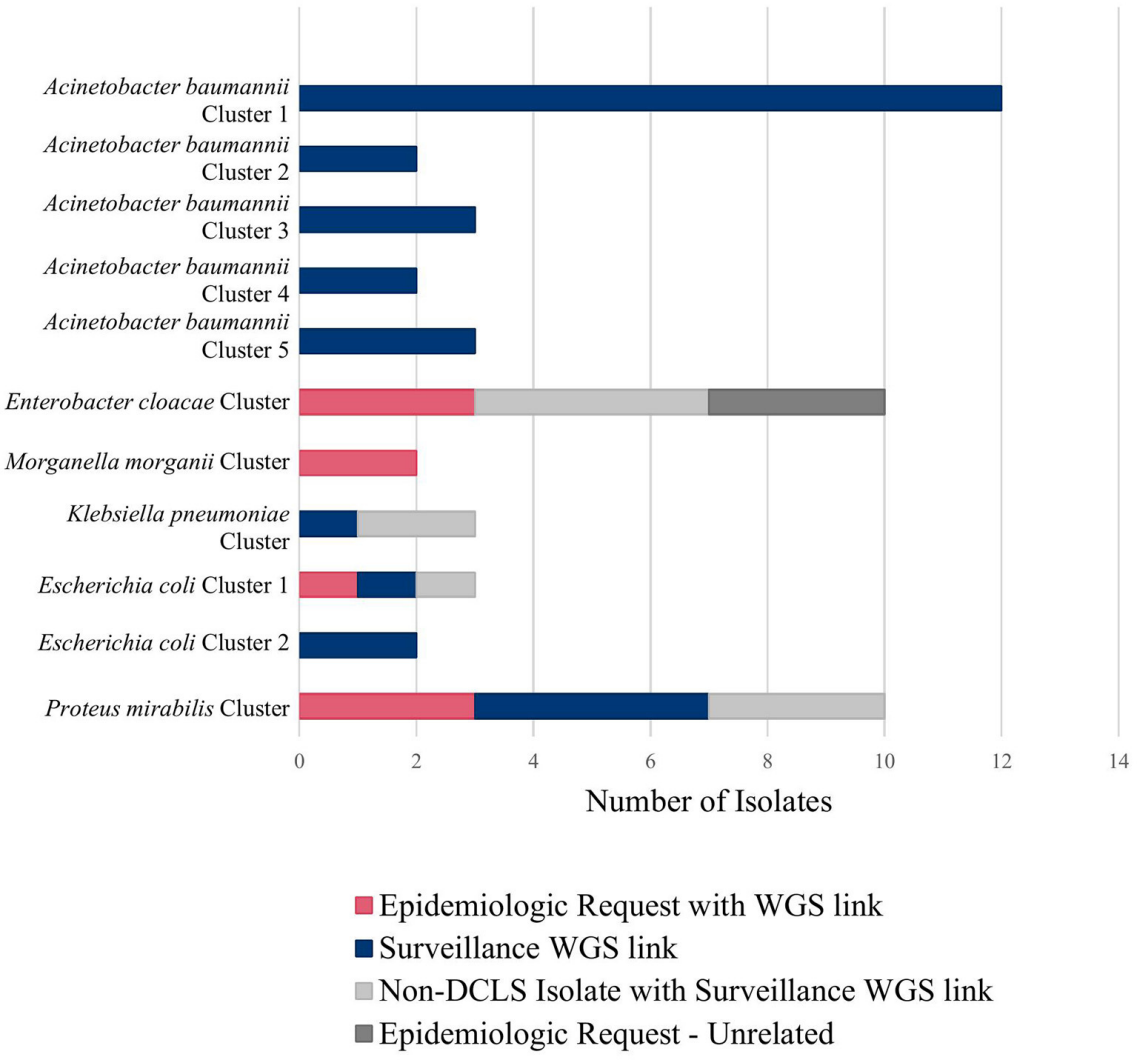
Clusters detected using tiered surveillance method (November 2021–April 2022).

For example, during an outbreak investigation of *Proteus mirabilis* isolates in Virginia for a local health department, the NCBI Pathogen Detection pipeline identified several other isolates sequenced by the Mid-Atlantic ARLN regional laboratory. Communication with the regional laboratory found that the isolates were colonization screening specimens sent from Virginia to the regional laboratory since DCLS does not currently perform colonization screening. Use of NGS for surveillance increased the number of isolates potentially related to this outbreak from 3 to 10 spanning a much longer period than originally investigated (Figure 2, *Proteus mirabilis* Cluster).

Figure 2 shows all the clusters meeting surveillance notification criteria during a pilot of the tiered surveillance method DCLS performed from November 2021 to April 2022. NGS surveillance provided many previously unidentified clusters and isolates (Shown in blue in Figure 2, Surveillance WGS link). As has been demonstrated by PulseNet for foodborne diseases, the ability to link cases of related infections using NGS is a powerful epidemiologic tool (29, 30). Surveillance and identification of related antimicrobial-resistant isolates provides an increased ability to respond and prevent the spread of this serious public health threat.

### Dryad/NCBI SNP discrepancies

Though rare, differences can occur in the SNP calls from the different pipelines. Several factors can cause discrepancies between these tools. Dryad is an open-source bioinformatics tool which lists each tool used and its version. NCBI Cluster Detection uses a suite of alternative tools curated by NCBI to perform assembly, genome annotation, antibiotic resistance determination and genome clustering (31, 32). Slight differences in tool heuristics and their parameters can lead to variations in SNP distances (33, 34). Reference genome selection can affect the SNP distances because the reference genome is the sequence to which all other cluster isolates are compared and if a more distant reference genome is used, there is a risk of losing genomic comparability for regions absent in the reference (35). The NCBI reference genome selection method chooses an in-group reference genome with the longest read from an initial dataset, which is often larger since it includes sequences from other NCBI submitters (31). Hickory selects the reference genome from within the user-defined dataset based on MASH distance (21). The user-defined dataset typically consists of isolates only sequenced and under suspicion of being outbreak-associated. Theoretically, there is an increased likelihood of identifying a greater number of SNPs because within dataset selected genomes should have a greater genetic identity. Masking portions of the genome sequence can also lead to differences in SNP distances. Some tools mask repetitive genome regions before SNP analysis, potentially altering downstream data. NCBI utilizes masking, while Dryad does not. Computing resources can also influence downstream analysis results (36). Masking and reference genome selection are the most likely causes of the significant discrepancies shown in Table 4.

**Table 4:** Dryad and NCBI discrepancies.

**Cluster**	**Isolate #**	**Dryad**	**NCBI**
*Proteus mirabilis*	2022EP-00001 & 7 isolate cluster	16-35 SNPs	4-10 SNPs
*Acinetobacter baumannii*	2021EP-00086 & 2021EP-00090	64-75 SNPs	7-14 SNPs
*Enterobacter cloacae*	2019EP-00005 & 2019EP-00121	479 SNPs	19 SNPs
*Klebsiella pneumoniae*	2022EP-00149 & 2022EP-00173	292 SNPs	18 SNPs

### NDM-19/NDM-7 cluster

Another benefit to running multiple analysis tools is using repeat results as a check to ensure the result report includes all the resistance genes present in the genome. On rare occasions, one AMR analysis tool doesn't report a gene found by another AMR prediction tool, and further analysis is required to verify the results. One example of when running two analysis tools proved beneficial was with an NDM *Klebsiella pneumoniae* outbreak investigation. On NCBI Pathogen Detection, AMR prediction of one isolate (2022EP-00101) had an NDM-19 gene, and the other isolate (2022EP-00107) had an NDM-7 gene. Dryad analysis results lacked the NDM-7 on 2022EP-00107 in the DCLS AMR report. Both isolates had positive NDM results from the Streck ARM-D, β-lactamase PCR kit further verifying the NCBI results. Re-sequencing and repeat Dryad analysis produced the same results. Combining the reads from both sequencing runs provided more depth and coverage, and Dryad analysis of the combined assembly identified the NDM-7 gene on the AMR prediction profile. Using more than one tool proved significant because results from one bioinformatics tool showed a gap in the results identified by the second tool, and further supported the overall SNP comparison indicating isolate genetic identity.

### AMR genes reporting

AMR gene predictions can produce a long list of resistance genes. Determining which genes to report to the epidemiologist has been a significant challenge. Including a list of all the genes identified can be overwhelming and not always informative or helpful since epidemiologists already have the antimicrobial susceptibility testing (AST) results, and prediction of a gene is not equivalent to expression. At DCLS, reporting AMR genes is based on the significance of the gene within the isolate or outbreak cluster, as determined by relevance to other phenotypic testing by mCIM, AST and PCR. A gene and allele number are always provided for carbapenemase genes since these genes are of interest to the CDC.

**Figure 3:**
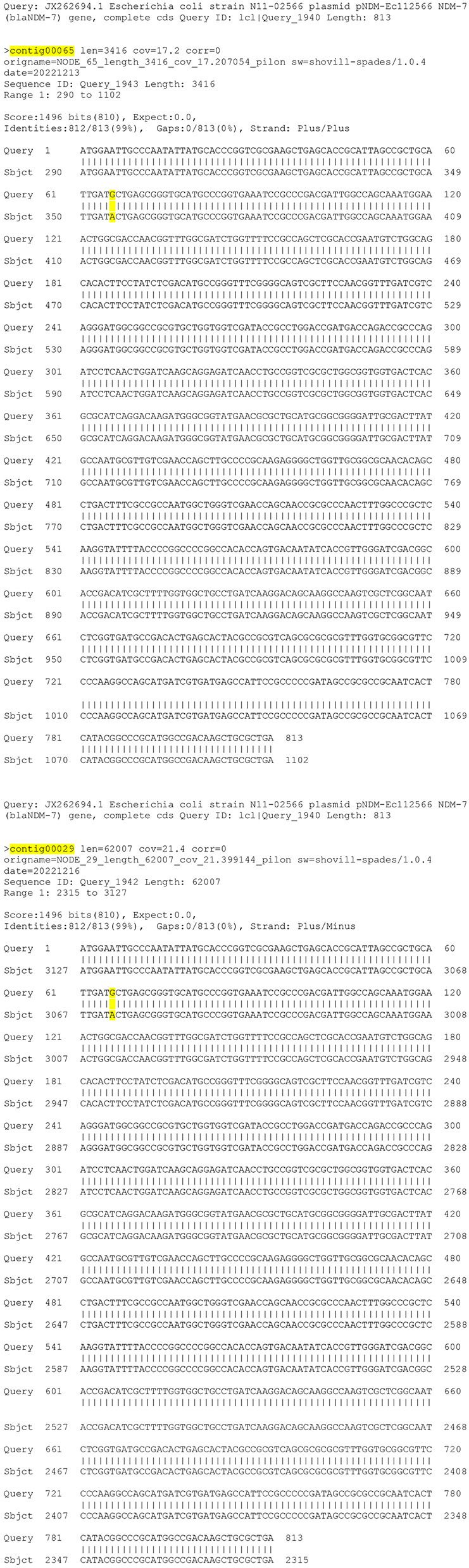
Gene mutation alignment.

For example, knowing an isolate or outbreak cluster carries the NDM-5 gene will provide specific information on which resistance gene is responsible for the carbapenem resistance. Allele identification also enables tracking of the frequency of individual alleles within a geographic area. If an allele is unknown, meaning the beta-lactamase (*bla*) gene is returned un-numbered by the AMR prediction method (ex: NDM-5 vs. NDM), further analysis is necessary to verify the presence of a unique allele and identify the responsible genome mutation (24). Using an alignment tool to compare the un-numbered gene sequence to the closest neighbor allows for identification of the nucleotide differences between the genes. Requesting an allele number for these un-numbered alleles is done through NCBI (37). Recently DCLS identified an un-numbered NDM allele that was a mutation of an NDM-7 in a *K. pneumoniae*. This gene had an M22I amino acid change due to a point mutation (Figure 3). The novel gene was named NDM-50 by NCBI. Surveillance detected two other isolates with this gene in Virginia over the next 2 months. Genome alignment can also be used to verify gene mutations of numbered alleles in closely related isolates as in the NDM-19/NDM-7 cluster above.

Currently, NGS result reports to epidemiologists include only genes indicated by PCR-detection. If an antimicrobial resistance gene other than a carbapenemase gene is predicted in one outbreak isolate, but absent in another, that could explain the difference in susceptibility results of a specific drug in the isolate's AST profiles, then a comment is added to the surveillance report shared with the health department. The report states that the difference in the AST profile is most likely due to the presence or absence of a resistance gene without naming the specific gene. The report may also include other novel genes causing carbapenem resistance identified in the organism of interest. Each report includes a disclaimer stating results are for epidemiologic purposes only and not for clinical diagnosis or patient management.

### Hypervirulence genes

The emergence of hypervirulent antimicrobial resistant bacteria has led to increased concern, as hypervirulence genes have been known to lead to more invasive and life-threatening illnesses. Hypervirulence and antimicrobial resistance were considered to be two divergent evolutionary pathways. However, in recent years organisms harboring both hypervirulence and antimicrobial resistance genes have emerged (38). Since DCLS added GAMMA to AMR analysis in February of 2022, of the 234 isolates analyzed the following hypervirulence genes were detected: 7 iroB-6, 3 iroB-23, 10 iucA-45, 1 iucA-18, 1 iucA-1, and 2 rmpA2–3. These genes were identified using a custom-database provided by CDC. These hypervirulence genes were all present in isolates that also tested positive for at least one carbapenem-resistance gene. Hypervirulence genes were originally described in *K. pneumoniae* isolates. However, the majority of the hypervirulence genes identified by DCLS since implementing GAMMA have been found in *E. cloacae* and *Escherichia coli*. One iroB-6 isolate was part of a multistate NDM + *E. cloacae* cluster found using the tiered method for surveillance.

## Discussion

NGS has provided a higher-resolution method for identifying and tracking resistance and hypervirulence genes of concern, as well as performing a critical role in the epidemiological investigations of *Candida auris* and carbapenamase-producing organisms. These techniques allow epidemiologists to study epidemiological links between microorganisms. The lack of a centralized national database for CRO genomic epidemiology has stymied proactive surveillance-based detection of possible clusters of interest across multiple facilities, temporally disparate cases, and prolonged time frames. The methodology described herein harnesses a publicly available data repository that provides centralized and integrated bacterial pathogen genomic comparisons for cluster prediction. Notification tools available through NCBI can alert laboratorians and epidemiologists to matches to jurisdictional isolates, as they are identified in the Pathogen Detection algorithm. Further interrogating putative clusters with a within data-set reference can help to further discern the extent of genomic differences which serve as a proxy for likelihood of transmission of a common infectious bacterial strain. NGS has been used many times to assist Virginia Department of Health epidemiologists in the quest to stop the spread of disease and antimicrobial resistance.

In 2019, an outbreak of KPC *Pseudomonas aeruginosa* in Southwest Virginia at an acute care hospital was investigated. To determine if there was spread within the facility, screening was conducted at the hospital as well as an infection prevention and control assessment. Epidemiologists found a total of 2 cases within the facility and determined there was no spread outside of the facility. The investigation was considered closed.

In September of 2022, another case of KPC *Pseudomonas aeruginosa* was discovered in the same acute care hospital and was believed to be an isolated case. When the epidemiologists received the NGS surveillance results, they were able to determine that the case was 0 SNPs apart from the 2 cases in 2019. This information shifted the investigation and encouraged the team to investigate the possibility of sustained reservoirs within the hospital itself.

The source of the outbreak has yet to be determined; the investigation is still ongoing. NGS has enabled the team of epidemiologists to gain insight into the linkage between these three cases, whereas before it was thought that the cases were unrelated. NGS gives epidemiologists an extra tool to be able to stop multi-drug resistant organisms and protect some of our most vulnerable populations. By using NGS to elucidate linkages across outbreaks and identify the presence of resistance genes adds another line of defense to the arsenal of public health.

This example demonstrates the value of adding NGS surveillance to the DCLS microbiology workflow. These results can be used by epidemiologists to improve the prevention and control of these highly resistant infectious pathogens. In addition, surveillance enables the tracking and identification of novel and emerging resistance genes and pathogens within our region. Limitations to surveillance using NGS include the dependence on hospital compliance to CRO submission laws. Funding also limits sequencing all CRO isolates which may leave gaps in tracking the spread of AMR. In addition, not all isolates are submitted to NCBI, and metadata can be lacking. Deidentification can prevent linking patients from Virginia tested in other states due to cross border healthcare or reference laboratory testing. Multistate outbreaks can be difficult for follow up due to multiple health departments and public health laboratories involvement. Lack of standardization of AMR tools and databases also inhibits comparison of results from one source to another. Despite the limitations, implementing NGS surveillance over the last year has improved awareness and understanding of carbapenem-resistant organisms and their resistance genes within Virginia for both the public health laboratory and the health department. This raised awareness has shown the need for plasmid genomics to track the spread of plasmids and resistant genes between bacterial species and is the focus of future work at DCLS.

## Data availability statement

The datasets presented in this study can be found in online repositories. The names of the repository/repositories and accession number(s) can be found below: https://www.ncbi.nlm.nih.gov/2022EP-00101, https://www.ncbi.nlm.nih.gov/2022EP-00107.

## Author contributions

KG and RS conceptualized the study, analysis plan, and analyzed the data. CD performed sequencing and wet lab data analysis. AO'R contributed to writing the draft and provided data. LT provided technical oversight and contributed to writing and editing the manuscript. All authors contributed to writing the final manuscript.
